# Binge drinking profile and sleep duration among Panamanian college professors and administrative workers

**DOI:** 10.47626/1679-4435-2024-1330

**Published:** 2025-09-09

**Authors:** Lourdes Luz Iribarren-Llorente, Luis Gabriel Rangel Caballero, Natalie Patricia Vásquez Mendoza, Linette del Carmen Govea Vásquez, Alba Liliana Murillo López

**Affiliations:** 1 Facultad de Salud, Universidad Metropolitana de Educación, Ciencia y Tecnología, Ciudad de Panamá, Panamá.; 2 Facultad de Cultura Física, Deporte y Recreación, Universidad Santo Tomás, Bucaramanga, Santander, Colombia. Funding: Universidad Metropolitana de Educación, Ciencia y Tecnología, Panama, and Universidad Santo Tomás, Colombia

**Keywords:** sleep duration, binge drinking, professor, university, tobacco use, duração do sono, consumo excessivo de bebidas alcoólicas, docentes universitários, tabagismo

## Abstract

**Introduction:**

Experts recommend that adults should regularly sleep at least 7 hours per night to benefit health and avoid negative outcomes. Binge drinking is the most common pattern of harmful alcohol consumption in the United States and is associated with injuries and diseases.

**Objectives:**

To investigate the association between sleep duration recommendations and binge drinking among Panamanian college professors and administrative workers.

**Methods:**

A cross-sectional study was conducted with 253 Panamanian professors and college administrative workers (171 women, 82 men) from four cities, affiliated with a private university, between June and December 2022. Descriptive, bivariate, and multivariate analyses were performed using logistic regression models. Data were collected according to the World Health Organization Noncommunicable Disease Risk Factor Surveillance method.

**Results:**

A total of 23.32% of participants reported binge drinking, while only 2.77% reported smoking. Men had a higher prevalence of binge drinking (p = 0.010) and smoking (p = 0.026) than women. Regarding sleep duration, 50.59% of participants did not meet the recommendation of 7 hours of sleep per night. After adjusting for sex, age, and socioeconomic level, binge drinking was associated with not meeting sleep duration recommendations (odds ratio: 2.049, 95%CI 1.0102-3.810, p = 0.023).

**Conclusions:**

Participants who reported binge drinking had a higher likelihood of not meeting the Joint Consensus Statement of the American Academy of Sleep Medicine and Sleep Research Society recommendations on sleep duration.

## Abstract (pt)

**Introdução:**

Especialistas aconselham que um adulto deveria dormir 7 horas por noite regularmente para obter benefícios para a saúde e evitar resultados negativos. O consumo excessivo de bebidas alcoólicas (*binge drinking*) é o padrão mais comum de consumo danoso de álcool nos Estados Unidos, e é associado com lesões e doenças.

**Objetivos:**

Estabelecer a associação entre as recomendações sobre a duração do sono e o consumo excessivo de bebidas alcoólicas em professores e trabalhadores administrativos universitários panamenhos.

**Métodos:**

Estudo transversal realizado em 253 professores e administrativos universitários panamenhos (171 mulheres, 82 homens) oriundos de 4 cidades em uma universidade no período junho-dezembro 2022. Análises descritivas, bivariadas e multivariadas foram realizadas usando modelos de regressão logística. Para a coleta de dados, aplicou-se o questionário básico do instrumento STEPS da Organização Mundial da Saúde.

**Resultados:**

23,32% dos participantes reportaram consumo excessivo de bebidas alcoólicas e 2,77% registraram tabagismo. Os homens tiveram uma prevalência maior de consumo excessivo de álcool (p = 0,010) e tabagismo (p = 0,026) do que as mulheres. Em relação à duração do sono, 50,59% dos participantes não cumprem as recomendações de dormir 7 horas cada noite. Depois de ajustar por sexo, idade e nível socioeconômico, o consumo excessivo de bebidas alcoólicas foi associado com as recomendações de duração do sono (*odds ratio*: 2,049, CI95% 1,0102-3,810, p = 0,023).

**Conclusões:**

Os participantes que reportaram consumo excessivo de bebidas alcoólicas tiveram uma probabilidade maior de não cumprir as recomendações da American Academy of Sleep Medicine and Sleep Research Society sobre a duração do sono.

## INTRODUCTION

Sleep is crucial for favorable health. According to the Joint Consensus Statement of the American Academy of Sleep Medicine (AASM) and Sleep Research Society (SRS), adults should regularly sleep 7 or more hours per night to benefit health. Sleeping less than the recommended amount is associated with negative health outcomes such as depression, overweight and obesity, hypertension, diabetes, heart disease, stroke, and a higher risk of death. This panel of experts also recommends that people concerned about sleeping too little should consult their health care provider.^1^

Globally, noncommunicable diseases (NCDs) are, according to the World Health Organization (WHO), the leading cause of death and disability. NCDs such as cardiovascular disease, cancer, diabetes, and chronic lung illnesses are collectively responsible for 5.5 million deaths every year in the Region of the Americas.^2^ In 2019, in Panama, the main causes of death for both sexes were ischemic heart disease and stroke.^3^ The WHO has identified harmful alcohol consumption, physical inactivity, unhealthy diet, and tobacco use as behavioral risk factors that increase the risk of developing NCDs.^2^

The WHO defines alcohol as a toxic and psychoactive substance that can lead to dependence. Alcohol consumption is linked to more than 200 diseases, injuries, and other health conditions.^4^ The Global Alcohol Action Plan 2022-2030 describes harmful use of alcohol as “drinking that causes detrimental health and social consequences for the drinker, the people around the drinker and society at large, as well as patterns of drinking that are associated with increased risk of adverse health outcomes”.^5^

According to the Centers for Disease Control and Prevention (CDC), binge drinking is the most common pattern of harmful alcohol consumption in the United States and is associated with injuries and diseases. Binge drinking is defined as the consumption of five or more drinks on one occasion for men and four or more drinks on one occasion for women in the last month.^6^ The global prevalence of binge drinking in the general population aged 15 years or older is 18.2%^7^; in the Region of the Americas, this prevalence is 21.3%^7^ and, specifically in Panama, the prevalence of binge drinking was 5.7% in 2013.^8^

Tobacco use is responsible for more than 8 million deaths each year and is considered one of the greatest public health problems in the world. In 2020, approximately 22.3% of the global population reported using tobacco (36.7% of men and 7.8% of women).^9^ In Panama, in 2023, 4.4% of the population reported using tobacco – the lowest prevalence in the Region of the Americas.^10^

There is scientific evidence supporting the association between sleep duration and binge drinking^11^-^13^ as well as tobacco use^14^,^15^ among adults worldwide. Despite the established link between sleep duration, binge drinking, and tobacco use in adults, there is limited data on this topic among college professors and administrative workers in Panama.

Considering this, the objective of this study was to investigate the association between sleep duration and binge drinking and tobacco use in Panamanian college professors and administrative workers. This information is crucial for designing strategies to promote healthy habits in this population, which could help improve both mental and physical health.

## METHODS

This was a cross-sectional study involving 253 Panamanian professors and college administrative workers (171 women, 82 men) from four cities, affiliated with a private university, conducted between June and December 2022. A convenience sampling method was used. Professors and administrative workers of legal age were invited to participate, and those who voluntarily agreed by signing the informed consent form were included. Pregnant workers did not participate in this study.

Sleep duration was the dependent variable. Professors and administrative workers were asked to report the average number of hours they actually slept each night during the past month. Based on the recommended sleep duration,^1^ participants were classified as “Not meeting the recommendations” if they reported sleeping less than 7 hours per night.

Binge drinking and current smoking were the independent variables. Five professionals (three physical therapists, one psychologist, and one specialist in physical culture, recreation, and sports) administered the Basic Questionnaire of the WHO STEPS Instrument (Step 1) at the facilities of the Universidad Metropolitana de Educación, Ciencia y Tecnología, Panama (UMECIT), following the WHO STEPS Surveillance Manual.^16^

Binge drinking was defined as the consumption of five or more drinks on a single occasion for men and four or more drinks on a single occasion for women in the last month; and current smoking was defined as the consumption of at least one cigarette in the past month.^16^ Other sociodemographic variables, such as sex, age, occupation, and socioeconomic level, were also considered.

This study was conducted in accordance with the Declaration of Helsinki and the guidelines of the Panamanian Ministry of Health (RESEGIS code 3215). It was approved by the Bioethics Committee of the UMECIT. Participants provided informed consent, which included a description of the study’s objective, the procedures to be conducted, the voluntary nature of participation, and assurance of data confidentiality. Additionally, participants were identified in the database using unique codes.

## DATA ANALYSIS

Descriptive characteristics of participants were analyzed. Categorical variables were expressed as frequencies and percentages. For quantitative variables, the type of data distribution was assessed using the Shapiro-Wilk test. Variables with a normal distribution were expressed as means and SD, while those without a normal distribution were expressed as medians and interquartile ranges.

To determine statistically significant differences by sex, the chi-square test, Fisher’s exact test, Student’s *t* test, and Mann-Whitney’s *U* test were used. Bivariate analyses were performed between sleep duration recommendations and each independent variable. Variables with p < 0.20 were included in the multivariate models.

Logistic regression models were employed using the dichotomous dependent variable (Meeting the recommendations/Not meeting the recommendations) to identify factors associated with the outcome of interest. Data collected through the aforementioned procedures were entered into an Excel database, which was then exported to the Stata version 12.1/IC statistical software to generate the results.

## RESULTS

Most participants were women (67.58%), belonged to a medium socioeconomic level (90.91%), and were professors (65.22%). The mean age of participants was 41.55 years (SD: 11.87). Among the total number of participants, 49.41% of professors and administrative workers met the recommendations for sleep duration (Table 1).

**Table 1:** General characteristics and sleep duration by sex

Characteristic	Total (n = 253)	Women (n = 171)	Men (n = 82)	p-value
Socioeconomic level, n (%)				
Low	20 (7.91)	17 (9.94)	3 (3.66)	0.094
Medium	230 (90.91)	153 (89.47)	77 (93.90)	
High	3 (1.19)	1 (0.58)	2 (66.67)	
Occupation, n (%)				
Professor	165 (65.22)	106 (61.99)	59 (71.95)	0.077
Administrative worker	88 (34.78)	65 (38.01)	23 (28.05)	
Recommended sleep duration,* n (%)				
Yes	125 (49.41)	79 (46.20)	46 (56.10)	
No	128 (50.59)	92 (53.80)	36 (43.90)	0.140
Age (years), mean (SD)	41.55 (11.87)	41.08 (11.74)	42.53 (12.14)	0.364
Hours of sleep per day, mean (SD)	6.50 (1.39)	6.45 (1.43)	6.63 (1.31)	0.327

* According to the Joint Consensus Statement of the American Academy of Sleep Medicine (AASM) and Sleep Research Society (SRS).

Almost a quarter of participants reported binge drinking. The percentage of men who reported binge drinking was higher than that of women, showing a statistically significant difference. Only 2.77% of the study population were current smokers. In contrast, most participants did not meet the recommended daily servings of fruits and vegetables (Table 2).

**Table 2:** Alcohol consumption and tobacco use by sex

Characteristic	Total (n = 253)	Women (n = 171)	Men (n = 82)	p-value
Binge drinking, n (%)				
Yes	59 (23.32)	32 (18.71)	27 (32.93)	0.010
No	194 (76.68)	139 (81.29)	55 (67.07)	
Consumption of alcohol in the past 30 days, n (%)				0.546
Yes	118 (46.64)	82 (47.95)	36 (43.90)	
No	135 (53.36)	89 (52.05)	46 (56.10)	
Current smoking, n (%)				
Yes	7 (2.77)	5 (2.92)	2 (2.44)	0.592
No	246 (97.23)	166 (97.08)	80 (97.56)	
Past (lifetime) smoking, n (%)				
Yes	31 (12.25)	23 (13.45)	8 (9.76)	0.267
No	222 (87.75)	148 (86.55)	74 (90.24)	
Total drinks per month, median (IQR)	0 (0-8)	0 (0-5)	0 (4-18)	0.380
Hours of sleep per day, mean (SD)	6.50 (1.39)	6.45 (1.43)	6.63 (1.31)	0.327

IQR = interquartile range.

After adjusting for sex, age, and socioeconomic level, participants who reported binge drinking had a higher likelihood of not meeting the recommended sleep duration (odds ratio [OR] 2.049; 95%CI 1.102-3.810; p = 0.023) (Table 3).

**Table 3:** Association between sleep duration, binge drinking, and tobacco use

Characteristic	OR crude			OR adjusted*		
	OR	95%CI	p-value	OR	95%CI	p-value
Sleep duration						
Binge drinking	1.735	0.957-3.143	0.069	2.049	1.102-3.810	0.023
Current smoking	0.380	0.072-2.001	0.254	0.382	0.072-2.025	0.258

* Adjusted for sex, age, and socioeconomic level.

## DISCUSSION

To our knowledge, this is the first study to analyze the association between sleep duration and binge drinking and tobacco use among Panamanian university professors and administrative staff.

Regarding sleep duration, half of professors and administrative workers in this study did not meet the recommended amount. This result is higher than the findings of Chaput et al.17 in Canada, in which one-third of adults reported sleeping less than the time recommended for optimal health. In this study, the mean number of hours slept per day was higher among men than women, which is consistent with findings from studies conducted among college professors in Colombia,^18^ elementary school teachers in Malaysia,^19^ and elementary school teachers in Brazil.^20^

The high prevalence of insufficient sleep found in this study is concerning, considering that inadequate sleep has become a global public health problem – not only due to its high prevalence and association with mortality and morbidity, but also because it can negatively affect the workplace and impair mental health, cognitive function, and productivity.^21^

Regarding binge drinking, 23.32% of participants in this study reported engaging in this unhealthy habit. This prevalence is higher than the global average,^7^ as well as the rates reported in the Region of the Americas^7^ and in Panama.^8^ In relation to sex, in this study, the percentage of men with binge drinking was higher than that of women, in line with the findings of the Global Status Report on Alcohol and Health, which indicates that women engage less often in binge drinking.^7^

One possible explanation for the higher prevalence of binge drinking among professors in this study may be occupational stress. A study conducted among Brazilian professors reported that increasing pedagogical and administrative demands in universities, with measurable and quantifiable parameters that professors are forced to achieve, contribute to feelings of exhaustion and create a competitive work environment that leads to occupational stress. In this context, alcohol use may be rationalized as a means to relax, boost energy, or relieve pain.^22^ Another study, conducted with Ecuadorian university professors, found that men experiencing higher stress level were more likely to consume alcohol compared to those with lower stress levels.^23^

The main finding of this study is the association between binge drinking and insufficient sleep, which is in accordance with existing scientific evidence demonstrating a link between alcohol consumption and sleep duration in adults.^24^ Studies involving college students and maritime academy cadets have also revealed that nights in which alcohol was consumed were followed by reported disturbances in sleep quality and quantity.^12^,^13^ There is also evidence of an association between binge drinking and insomnia among Brazilian college students of health sciences.^25^

Scientific evidence has determined the effects of binge drinking on sleep. Banerjee highlights that alcohol acts as a central nervous system depressant and interacts with several neurotransmitter systems that are crucial for sleep regulation.^26^ Consuming large quantities of alcohol before sleep reduces sleep onset latency and alters sleep architecture early in the night, when blood alcohol levels are high, resulting in disrupted and poor-quality sleep later during the night.^27^

This study has some limitations. Only professors and administrative workers present at the university on the day of data collection were included. The sample represents a convenience subset of the university population. Another limitation is the cross-sectional design, which precludes conclusions about causality of the variables – only associations could be assessed. Finally, sleep questions used in population health questionnaires are susceptible to overestimating actual sleep duration when compared to objective measures.^28^

## CONCLUSION

In conclusion, half of participants in this study did not meet the recommendations for sleep duration, and almost a quarter reported binge drinking. Participants who reported binge drinking had a higher probability of not meeting the Joint Consensus Statement of the AASM and SRS recommendations on sleep duration.

Further studies using probabilistic sampling methods are needed to validate the findings of this study. These results support the development of strategies aimed at reducing binge drinking levels and promoting the importance of meeting recommended sleep duration, with the goal of improving both mental and physical health in this population.
